# Reply to Gorelick and Kunst: Paying reviewers will increase publication costs and could exacerbate inequities

**DOI:** 10.1073/pnas.2628160123

**Published:** 2026-10-16

**Authors:** Angela Moles, Stephen Bonser, Nigel Andrew, Daniel Falster, Russell Bonduriansky

**Affiliations:** ^a^ Evolution and Ecology Research Centre, School of Biological, Earth and Environmental Sciences, UNSW Sydney, Sydney, NSW 2052, Australia; ^b^ Faculty of Science and Engineering, Southern Cross University, Northern Rivers, NSW 2480, Australia

Gorelick and Kunst (1) suggest paying reviewers for their work is a better solution to the peer review crisis than a credit system such as Reciprocity (2). Paid reviewing is appealing to many, and addresses several problems with the current peer review system (3). However, paying reviewers has downsides that are avoided by a credit system.

First, the costs of paying reviewers will inevitably be borne by authors or their institutions. Biology Open paid reviewers £220 per manuscript, with a total additional cost in the second year of operation (i.e., after the costs of establishing the system) of £693 to £760 per manuscript (3). Nonprofit organizations like Biology Open’s publisher will presumably have no option but to pass these costs on to authors once the trial phase concludes. There seems to be some belief in the scientific community that for-profit publishers will absorb these costs. However, economic literature suggests that much of the increase will be passed through to customers (4, 5). Reviewing fees could be paid upfront, or only for accepted papers. Upfront fees may change the way authors respond to negative reviews. However, if accepted authors covered reviewing for unsuccessful submissions, the cost would be substantial (e.g., with 20% acceptance and £700/submission, authors would need to pay £3,500 per accepted manuscript on top of existing publication charges).

Paid peer review could exacerbate inequality. A uniform fee has unequal effects because it takes a larger proportion of income from those with fewer resources (6). Thus, increasing author charges adds to the disadvantages of underresourced researchers, institutions, and countries. Fee waivers help but are imperfect (7). Unequal access to reviewing invitations will also contribute to financial inequality. Further, treating reviewing as optional paid outside work raises ethical questions about conducting reviews as part of a salaried position and may not be allowed by some employers. Under Reciprocity, reviewing is simply part of the time-cost of doing research.

Paid peer review creates problematic incentives. For those in need of cash, payments provide a strong incentive to accept every review invitation, even if the manuscript is outside their area of expertise or they lack the time to do a thorough review. Conversely, for well-off researchers (e.g., senior researchers in wealthy countries), the offer of a small cash payment will provide little incentive to review (particularly after income tax). In an ideal system, everyone from experienced senior researchers to early career researchers worldwide would contribute to peer review, which would benefit from a diversity of perspectives. Reciprocity creates a more universal incentive to review, and less opportunity to game the system.

In summary, while paying peer reviewers is superficially appealing, it has substantial downsides. We believe a credit system that incentivizes everyone to contribute reviews in proportion to their paper submissions is the fairest option. However, both systems could operate simultaneously, as long as credits were still required for submissions so that wealthier researchers do not buy their way out of reviewing.

## Data Availability

There are no data underlying this work.

## References

[1] D. A. Gorelick, J. Kunst, Compensate peer reviewers; don’t conscript them. Proc. Natl. Acad. Sci. U.S.A. **123** (2026). 10.1073/pnas.2619714123 PMC13715780 42886757

[2] A. Moles , A transparent universal credit system to incentivize peer review. Proc. Natl. Acad. Sci. U.S.A. **123**, e2506681123 (2026). 10.1073/pnas.2506681123 PMC13168533 42090247

[3] D. A. Gorelick, A. Clark, Expanded implementation of Fast & Fair paid peer review reduces time to first decision without reducing review quality in a biology journal. bioRxiv [Preprint] (2026). 10.64898/2026.06.02.729548 (Accessed 9 August 2026).

[4] G. Deltas, M. Polemis, Price pass-through dependence on the source of cost increases: Evidence from the European gasoline market. Rev. Ind. Organ. **65**, 595–626 (2024).

[5] E. G. Weyl, M. Fabinger, Pass-through as an economic tool: Principles of incidence under imperfect competition. J. Polit. Econ. **121**, 528–583 (2013).

[6] L. Bing, B. Pettit, I. Slavinski, Incomparable punishments: How economic inequality contributes to the disparate impact of legal fines and fees. RSF **8**, 118–136 (2022). 10.7758/rsf.2022.8.2.06 PMC9030976 35463483

[7] U. G. Gardner Jr. , Article processing charge waiver policies as a barrier to oncology scholarship in low- and lower-middle-income countries. JCO Glob. Oncol. **7**, 1413–1417 (2021). 10.1200/GO.21.00143 PMC8478385 34554813

